# From Genomic Diagnosis to Personalized RNA Medicine: Advances in Next-Generation Sequencing and N-of-1 Antisense Oligonucleotide Therapies for Rare Genetic Diseases

**DOI:** 10.3390/genes17030318

**Published:** 2026-03-15

**Authors:** Paris Rodriguez Carstens, Hidenori Moriyama, Toshifumi Yokota

**Affiliations:** Department of Medical Genetics, Faculty of Medicine and Dentistry, University of Alberta, Edmonton, AB T6G 2H7, Canada

**Keywords:** next-generation sequencing, antisense oligonucleotides, N-of-1, rare genetic diseases

## Abstract

Next-generation sequencing (NGS) and antisense oligonucleotide (ASO) technologies are converging to transform the diagnosis and treatment of rare monogenic disorders. NGS enables comprehensive, single-test molecular diagnoses through targeted panels, whole-exome sequencing, and whole-genome sequencing, which together reveal pathogenic variants across coding, intronic, and structural domains. Integration with transcriptomic analyses, including RNA sequencing, further refines genotype–phenotype correlations and identifies splicing aberrations amenable to correction by ASOs. Therapeutic advances now span RNase H1-dependent gapmers for transcript knockdown, splice-modulating phosphorodiamidate morpholino oligomers (PMOs), and peptide/antibody-conjugated PMOs that enhance muscle and cardiac delivery. These platforms underpin the rise in N-of-1 ASO therapies—customized drugs developed for individual patients with unique pathogenic variants. Landmark cases such as Milasen and Atipeksen illustrate the clinical feasibility and ethical complexities of personalized RNA therapeutics, while updated FDA guidance supports expedited, patient-specific investigational pathways. Despite progress, challenges persist in delivery efficiency, long-term efficacy, and equitable access. Emerging approaches—including long-read sequencing, AI-driven oligo design, and improved delivery—promise to extend ASO precision and reach. This review synthesizes current advances linking genomic diagnosis to individualized RNA-targeted interventions, outlining how integrated NGS-ASO pipelines are reshaping the therapeutic landscape for rare genetic diseases.

## 1. Introduction

### 1.1. Rare Genetic Diseases

Rare diseases are defined in Europe and Canada as conditions affecting no more than 5 in 10,000 individuals. To date, between 6000 and 8000 rare diseases have been identified, of which approximately 80% have a genetic origin and 50–75% present during childhood. These disorders are typically chronic, progressive, and debilitating, contributing significantly to morbidity and mortality in affected populations [1]. Among the more common rare diseases is Duchenne muscular dystrophy (DMD), which affects roughly one in every 5000 males worldwide [2]. However, the rarity and phenotypic heterogeneity of such disorders make them challenging to diagnose, often resulting in diagnostic delays or misclassification [3]. Moreover, there remains no universal definition of a rare disease across jurisdictions, further complicating clinical trial design, treatment approval, and policy development. 

Recognizing these challenges, Rare Diseases International issued a landmark 2019 report calling for universal health coverage policies that explicitly include rare diseases. The United Nations subsequently recognized people living with rare diseases as a marginalized population, emphasizing their inclusion in global and national healthcare planning frameworks. These developments underscore an emerging global commitment to equitable access to diagnosis and treatment for individuals with rare diseases [4].

Concurrently, advances in Next-generation sequencing (NGS) have transformed the diagnostic landscape by enabling comprehensive molecular characterization through targeted panels, whole-exome sequencing (WES), and whole-genome sequencing (WGS). These approaches have drastically shortened the diagnostic odyssey and now provide the foundation for precision therapeutic development, linking genomic diagnosis directly to individualized RNA-based interventions.

### 1.2. From Diagnosis to Design

Advances in NGS have transformed the diagnostic landscape for rare genetic diseases, particularly neuromuscular disorders (NMDs) [5]. NGS enables high-throughput, genome-wide analysis that shortens diagnostic latency, increases accuracy, and reduces overall testing costs. Because many single-gene defects produce overlapping clinical phenotypes, NGS has become the first-line molecular diagnostic modality for NMDs, capable of identifying causal variants through a single, comprehensive assay [6]. Clinicians typically begin with targeted gene panels, which interrogate curated sets of disease-relevant genes selected according to clinical presentation. These panels vary in size and composition depending on the suspected category—such as myopathy, neuropathy, or muscular dystrophy—but generally offer rapid turnaround and high sensitivity for known genes [1,5].

Once a pathogenic variant is identified, antisense oligonucleotide (ASO) therapeutics can, in some cases, be rationally designed to correct the underlying RNA defect, though not all variants are amenable to such correction. ASOs are short, synthetic nucleic acid polymers that modulate gene expression via two main mechanisms [7]. RNase H1-dependent gapmers hybridize to target mRNA, recruit RNase H1, and trigger degradation of the transcript, resulting in selective gene silencing. Steric-blocking ASOs were initially developed to inhibit translation or destabilize target transcripts by physically obstructing ribosomal assembly, interfering with polyadenylation signals, or blocking regulatory RNA motifs. Subsequent advances extended their use to splice modulation, where ASOs bind to pre-mRNA and alter splice-site recognition to promote exon skipping or inclusion, thereby restoring or modifying protein expression [8]. Together, these mechanisms enable diverse therapeutic strategies—from transcript silencing in gain-of-function disorders to reading-frame restoration in loss-of-function diseases such as DMD and spinal muscular atrophy (SMA). However, the feasibility of ASO therapy depends on the variation class, transcript accessibility, and delivery efficiency, limiting its applicability to select pathogenic variants [7,8].

**Figure 1:**
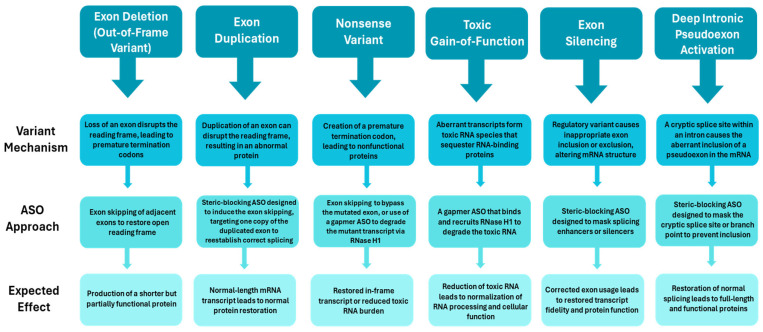
Schematic diagram showing ASO approaches for genetic variations.

The NGS-to-ASO pipeline is thus progressing from comprehensive genetic diagnosis, through variant-specific design of gapmer or splice-modulating ASOs, to the development of personalized therapies for previously untreatable disorders (Figure 1). This review highlights the recent advances linking genomic diagnostics to individualized RNA-targeted interventions, emphasizing their translational implications for rare genetic diseases.

## 2. NGS for Therapy Selection in Rare Diseases

### 2.1. NGS Assays and Detection

NGS is essential for the accurate identification of disease-causing variations and the implementation of precision diagnostics. Among NGS modalities, targeted gene panels are the most cost-effective option, analyzing a curated subset of genes relevant to a specific phenotype [9]. These panels provide high sequencing depth, rapid turnaround, and minimal incidental findings. Comprehensive neuromuscular panels can include several hundred genes associated with inherited myopathies and neuropathies. Consequently, targeted NGS is widely regarded as the preferred first-tier test for many NMDs, offering an optimal balance between diagnostic yield, cost, and clinical utility [10]. However, panel content varies among laboratories, and these assays cannot detect variants in genes not included in the curated list or in non-coding regions. Although diagnostic yields of 50–60% have been reported in selected NMD cohorts, limitations remain for detecting deep intronic, regulatory, or large structural variants (SVs) [11].

When targeted panels are inconclusive or when the phenotype is broad, WES is typically employed. WES captures the protein-coding portion of the genome—approximately 1–2% of total DNA—where the majority of pathogenic variants reside [10,11]. This approach enables both gene discovery and detection of novel or de novo variants beyond the constraints of predefined panels. However, WES may miss pathogenic changes in non-coding, deep intronic, or poorly captured regions, as well as certain SVs and repeat expansions [12]. It is therefore most effective for genetically heterogeneous conditions or after negative targeted testing.

WGS extends the tested coverage to nearly the entire genome, including both coding and non-coding regions, improving detection of complex SVs, copy-number changes, and repeat expansions. WGS interrogates both nuclear and mitochondrial DNA and yields more uniform coverage compared to WES [13]. Systematic reviews indicate that WGS increases diagnostic yield slightly over WES—approximately 38.6% vs. 37.8% across pediatric cohorts [14]. In NMDs, WGS has been used to identify deep intronic variants, such as pathogenic cryptic splice-site variants in *IGHMBP2* responsible for spinal muscular atrophy with respiratory distress type 1 (SMARD1) [15]. While WGS provides the most comprehensive view of genomic variation, it remains more expensive, generates complex data requiring significant bioinformatic support, and can yield a higher rate of variants of uncertain significance [16].

RNA sequencing (RNA-seq) complements DNA-based assays by revealing transcriptomic consequences of genomic variants. It profiles gene expression, splicing, and allele-specific expression from disease-relevant tissue (e.g., skeletal muscle), thereby providing mechanistic evidence for variant pathogenicity. RNA-seq is particularly valuable when DNA testing yields uncertain or monoallelic findings. In practice, RNA-seq has elucidated cryptic splicing defects in *DMD* and *COL6A3*, resolving previously undiagnosed cases of muscular dystrophy. However, the method requires high-quality RNA from relevant tissues and careful sample handling to preserve integrity [17].

Additional copy-number assays such as multiplex ligation-dependent probe amplification (MLPA) and comparative genomic hybridization (CGH) remain essential adjuncts, especially for genes such as *DMD*, *TTN*, or *NEB*, where large deletions or duplications account for the majority of pathogenic variants [11,17]. Even in the NGS era, these complementary techniques are recommended to ensure reliable detection and confirmation of exon-level copy-number changes.

In summary, the various NGS modalities provide distinct but complementary insights. Together, integrated genomic and transcriptomic analyses refine target selection, improve variant–therapy matching, and enhance the design and assessment of personalized ASO therapeutics.

### 2.2. Mapping Variant Classes to ASO Strategies

The mechanisms of ASO action fall broadly into two therapeutic categories: transcript knockdown and splicing modulation. In toxic gain-of-function disorders—where mutant transcripts or proteins exert deleterious effects—ASOs can induce selective degradation of the mRNA through RNase H1-mediated cleavage. These “gapmer” ASOs have been particularly successful in neurological disorders. A prominent example is SOD1-associated amyotrophic lateral sclerosis (ALS), where ASO-mediated suppression of mutant SOD1 transcripts reduces toxic protein accumulation [18]. Notably, in 2023, the FDA granted accelerated approval to tofersen for the treatment of SOD1-ALS, marking it as the first ASO therapy approved for this devastating neurodegenerative disease [19]. 

In contrast, loss-of-function disorders often benefit from splicing modulation strategies. Splice-switching ASOs can be designed to skip or include specific exons, thereby restoring the open reading frame or correcting aberrant splicing. Exon-skipping ASOs targeting DMD exon 51, 53, or 45 restore dystrophin translation by bypassing frameshift variants, producing a truncated protein similar to the Becker muscular dystrophy phenotype [20]. Alternatively, exon inclusion ASOs can restore functional transcripts when disease results from functional protein deficiency due to alternative splicing, as exemplified by nusinersen in spinal muscular atrophy (SMA), which promotes inclusion of exon 7 in SMN2 transcripts to increase SMN protein levels [21].

A third approach, allelic upregulation, is applicable to haploinsufficient genes using platforms such as TANGO (Targeted Augmentation of Nuclear Gene Output). In this strategy, ASOs block non-productive splicing events to shift the balance toward productive mRNA, thereby enhancing wild-type transcript expression. This mechanism has been validated in human genes with characterized non-productive isoforms [22].

### 2.3. Assay Limitations and Solutions

Despite substantial technological progress, several classes of pathogenic variants remain challenging to detect using standard short-read NGS.

Deep intronic variants represent a major limitation, as they often escape detection by WES or panel-based assays that capture only exons and adjacent boundaries. These variants can activate cryptic splice sites or introduce pseudo-exons, leading to aberrant transcripts [23,24]. Such variants are ideal candidates for splice-modulating ASOs, which can suppress cryptic exon inclusion. Their detection typically requires a combination of WGS, which provides full intronic coverage, and RNA-seq, which reveals corresponding splicing defects at the transcript level. For example, a deep intronic *IGHMBP2* variant underlying SMARD1 was identified by WGS and functionally confirmed by cDNA analysis [15]. Similarly, RNA-seq of muscle tissue has revealed DMD pseudo-exons missed by DNA-based tests [17].

SVs—including large deletions, duplications, inversions, and complex rearrangements—are another source of missed diagnoses. While WGS improves SV detection, highly repetitive or GC-rich regions and repeat expansions remain problematic for short-read sequencing [12]. A large neuromuscular genome-sequencing study reported that approximately one-third of diagnostic findings involved SVs or repeat expansions undetected by prior exome testing [25]. Emerging long-read sequencing technologies (e.g., Oxford Nanopore and PacBio) now offer improved resolution of complex SVs and tandem repeats, identifying clinically relevant variants missed by short-read methods [26]. 

Repeat expansion disorders pose specific challenges because short reads cannot reliably align across long, repetitive regions. Dedicated repeat-expansion assays or long-read WGS are required for accurate sizing and detection [27].

Finally, mitochondrial genome and tissue-specific expression issues can also elude standard NGS approaches. Many gene panels and WES pipelines omit the mitochondrial genome, despite its relevance to neuromuscular pathology. WGS, which includes both nuclear and mitochondrial DNA, or dedicated mtDNA sequencing, is therefore preferred in suspected mitochondrial disease [14,28]. Likewise, RNA-seq from disease-relevant tissue (e.g., skeletal muscle) can uncover pathogenic splicing events or allelic imbalances not observable in blood-derived samples.

## 3. Antisense Oligonucleotide Therapies

### 3.1. Mechanisms of ASO Therapies

ASOs are short, synthetic nucleic acid polymers designed to modulate gene expression at the RNA level. Rather than introducing an exogenous gene copy—as in gene therapy—ASOs directly target endogenous transcripts to restore, suppress, or fine-tune RNA function [29].

Historically, steric-blocking ASOs were the first to be developed. They act by physically obstructing access of cellular machinery—such as ribosomes, RNA-binding proteins, or splicing factors—to specific RNA motifs. Early applications used this approach for translational inhibition and transcript destabilization. Subsequent refinements showed that steric blocking could also modulate pre-mRNA splicing, establishing the foundation for splice-switching therapeutics [29,30]. In the 1990s, researchers demonstrated that steric blocking could redirect splicing in DMD myotubes, establishing the conceptual basis for splice-modulating therapy [31]. This breakthrough remains one of the most clinically validated ASO strategies.

Splice-switching ASOs function by masking or exposing specific pre-mRNA motifs such as splice sites, enhancers, or silencers. In DMD, exon-skipping ASOs target exons 45, 51, 53, and others to restore the reading frame and enable production of a truncated yet functional dystrophin protein—mimicking the milder Becker phenotype [32]. Similarly, exon-inclusion ASOs can correct exon-loss variants, as exemplified by nusinersen, which promotes inclusion of exon 7 in *SMN2* transcripts to restore functional SMN protein in SMA [21].

As ASO chemistry evolved, a second mechanistic class emerged in the 1980s—RNase H1-dependent “gapmers”. These oligonucleotides contain a central DNA segment flanked by chemically modified ribonucleotides (e.g., 2′-O-methoxyethyl or 2′-O-methyl groups) to enhance affinity and nuclease resistance. When hybridized to a target RNA, the DNA–RNA duplex recruits RNase H1, which cleaves the RNA strand and triggers selective transcript degradation [29]. This mechanism has been successfully applied to diseases driven by toxic gain-of-function transcripts, such as SOD1-associated ALS [29].

Chemical optimization has underpinned the success of both splice-modulating and knockdown ASOs. The phosphorodiamidate morpholino oligomer (PMO) backbone—featuring a morpholine ring and charge-neutral phosphorodiamidate linkage—provides exceptional biostability and low off-target activity [33]. However, its neutral charge limits cellular uptake, especially in cardiac and skeletal muscle. To address this, peptide-conjugated PMOs (PPMOs) were developed, in which a cell-penetrating or muscle-targeting peptide such as DG9 is covalently linked to the PMO backbone. This modification enhances uptake and exon-skipping efficiency in both skeletal and cardiac tissue, restoring dystrophin expression and improving muscle function in DMD models [34].

### 3.2. Delivery Strategies

While ASO chemistries have advanced substantially, effective delivery to target tissues remains one of the principal challenges in translating RNA therapeutics into durable clinical benefit. ASOs are large, polar macromolecules that do not readily cross cell membranes or biological barriers; thus, their biodistribution, route of administration, and chemical design must be carefully optimized for each indication [35].

Early exon-skipping studies in DMD relied on local intramuscular injection of PMOs, achieving robust exon skipping in treated muscle fibers but limited systemic exposure. Subsequent preclinical progress demonstrated that systemic intravenous administration could achieve body-wide delivery, albeit at high doses due to rapid renal clearance and endosomal trapping [35]. The clinically approved PMO drugs for DMD—eteplirsen, golodirsen, viltolarsen, and casimersen—all use weekly intravenous infusion to maintain sufficient plasma and muscle concentrations for therapeutic exon skipping [36].

However, biodistribution remains uneven. Skeletal muscle uptake is typically greater than that in cardiac muscle, largely due to differences in vascular permeability, perfusion, and endosomal escape efficiency. This discrepancy limits the treatment of DMD cardiomyopathy, which is a major cause of mortality [37].

To overcome this, next-generation chemistries such as PPMOs were developed. By attaching a cell-penetrating or muscle-targeting peptide (e.g., DG9), PPMOs enhance cellular internalization and nuclear delivery, producing markedly higher exon-skipping efficiency and dystrophin restoration in both skeletal and cardiac tissues [34,38]. Alternative conjugation and carrier systems are being investigated to improve delivery precision and durability. These include antibody-ASO conjugates, GalNAc ligands for liver targeting, lipid and polymeric nanoparticles, and antibody-masked ASOs designed for controlled tissue release [39,40,41].

For diseases affecting the central nervous system (CNS), such as SMA or Batten disease, intrathecal delivery is the preferred route, allowing direct administration into the cerebrospinal fluid (CSF) and bypassing the blood–brain barrier [42]. Nusinersen and the personalized ASO Milasen are delivered intrathecally to achieve high CNS exposure with relatively low systemic toxicity. However, intrathecal dosing is invasive, requires clinical monitoring, and provides limited peripheral distribution, making it less suited for systemic neuromuscular disorders [42,43].

A persistent challenge is the transient nature of ASO activity. Because ASOs act on RNA without altering the genome, their effects wane as molecules are degraded or diluted during cell turnover. Most current therapies therefore require chronic administration—weekly infusions for PMOs, or quarterly intrathecal dosing for CNS-directed ASOs [43]. Long-acting formulations, nanoparticle encapsulation, and peptide or antibody conjugation offer promising approaches to extend half-life and maintain therapeutic efficacy with less frequent dosing [44].

### 3.3. Case Studies: N-of-1 and Platform-Based ASO Development

The practical realization of personalized antisense therapy began only recently but has rapidly reshaped translational paradigms. While the concept of individualized ASO design had long been envisioned, its feasibility was first demonstrated through pioneering N-of-1 cases, in which bespoke oligonucleotides were conceived, validated, and administered within an unprecedented timeframe. These cases illustrate how the convergence of genomic diagnosis, modular chemistry, and adaptive regulation has transformed rare-disease drug development from a population-based to a patient-specific endeavor.

#### 3.3.1. Milasen: Rapid Customization for Batten Disease

The landmark example is Milasen, designed in 2017 for a child with *CLN7 (MFSD8)*-associated Batten disease. WGS revealed a deep intronic insertion that created a cryptic splice acceptor, producing a pseudo-exon in *MFSD8* transcripts. Within months, researchers at Boston Children’s Hospital designed a steric-blocking 2′-O-methoxyethyl ASO to mask the aberrant splice site, restoring normal splicing in patient fibroblasts. Preclinical toxicology and manufacturing were completed under The U.S. Food and Drug Administration (FDA) guidance in less than a year, and intrathecal administration led to measurable molecular and clinical improvement [45,46]. Although Milasen significantly reduced seizure frequency, it could not reverse the pre-existing visual loss and neurological decline that had occurred prior to treatment. This outcome underscored the inherent limitations of post-symptomatic intervention in rapidly progressive neurodegenerative disorders. Milasen thus provided proof that individualized RNA therapeutics could be designed, tested, and deployed safely under existing regulatory frameworks.

#### 3.3.2. Valeriasen: Targeted Knockdown in KCNT1-Related Epileptic Encephalopathy

Building on this precedent, Valeriasen was developed for a child with epilepsy of infancy with migrating focal seizures caused by a *KCNT1 c.1421A>G* gain-of-function variant. *KCNT1* encodes the sodium-activated potassium channel KNa1.1, whose hyperactive currents produce severe, drug-resistant seizures. Using prior preclinical data showing *KCNT1* suppression via RNase H-dependent gapmers, researchers designed a patient-specific gapmer ASO to selectively degrade the mutant transcript. The sequence was optimized and validated in vitro, followed by rapid 10-week toxicology and manufacturing. Intrathecal administration began in 2020 under a dose-escalation protocol. Although Valeria’s course was complicated by hydrocephalus—a risk shared across multiple CNS-delivered ASO contexts—the study demonstrated that allele-specific silencing could be executed as a fully individualized intervention. Regrettably, Valeria passed in 2021 due to hydrocephalus, which has been reported in patients treated with ASOs intrathecally for other diseases, as well as a joint symptom with epilepsy of underlying neurological conditions. Currently, a revised version of valeriasen is being developed to treat *KCNT1* pathogenic variants [47].

#### 3.3.3. Atipeksen: Splice Correction for Ataxia-Telangiectasia

The third exemplar, Atipeksen, extended the N-of-1 paradigm to ataxia-telangiectasia (A-T), a neurodegenerative disorder caused by biallelic *ATM* pathogenic variants. Kim et al. (2023) established a systematic framework for individualized splice-switching ASO development and applied it to a patient (Ipek) carrying a 13-bp intronic deletion (*c.*8585-13_8598del) on one allele and a *c.*7865C>T splice-site variant on the other [48]. Thirty-two candidate 2′-O-methoxyethyl phosphorothioate ASOs were screened in patient fibroblasts; the optimal sequence, AT008, restored up to 50% of normal *ATM* splicing and was designated Atipeksen. Intrathecal administration began at 2 years 9 months of age, escalating from 3.5 mg to 42 mg over 10 weeks, then maintained every 8–12 weeks. Over three years of follow-up, Ipek’s neurological scores remained milder than typical for A-T progression, suggesting disease stabilization. The study demonstrated the reproducibility of the Milasen-style development pipeline and underscored the importance of early intervention before irreversible neurodegeneration [48].

#### 3.3.4. Toward Scalable Personalization

Collectively, Milasen, Valeriasen, and Atipeksen exemplify how NGS, ASO chemistry, and translational infrastructure now intersect to deliver rapid, patient-specific therapies. Each case leveraged prior toxicology data and standardized manufacturing to shorten timelines from variant discovery to first-in-human dosing. These experiences catalyzed the establishment of platform-based frameworks that reuse validated chemistries, dosing regimens, and regulatory templates—enabling future N-of-few programs addressing small patient clusters with shared mechanistic variants. Ethical and logistical challenges remain, but these early exemplars have transformed N-of-1 therapy from an experimental exception into an emerging, replicable model of interventional genetics [49].

## 4. N-of-1 Therapies

The emergence of ASO therapeutics has redefined individualized medicine, enabling development of N-of-1 treatments for patients with unique pathogenic variants. In these cases, ASOs are custom-designed, manufactured, and clinically implemented for a single individual, bridging rapid genomic diagnosis with tailored RNA-level correction. While this approach provides new hope for ultra-rare, life-limiting disorders, it also raises complex scientific, ethical, and logistical questions concerning feasibility, regulation, and equitable access [49].

### 4.1. Regulatory Evolution and Foundational Cases

The development of Milasen for CLN7-related Batten disease established both the technical and regulatory precedent for N-of-1 therapy. FDA subsequently issued its first draft guidance for individualized ASO Investigational New Drug (IND) applications in 2021 (FDA-2021-D-1140). This document introduced a flexible, case-by-case framework allowing expedited review while maintaining rigorous safety and manufacturing oversight [50].

Key provisions include tailored safety monitoring based on ASO pharmacokinetics, frequent platelet surveillance to mitigate phosphorothioate-related thrombocytopenia, and mandatory inpatient observation during initial dosing. Chemistry, Manufacturing, and Controls sections must specify oligo structure, synthesis flow, purity, and stability, ensuring proportional compliance with current Good Manufacturing Practice (GMP) despite accelerated timelines. These measures enable safe, patient-specific implementation without compromising scientific or ethical standards [50,51].

Within this newly articulated regulatory framework, subsequent cases such as Valeriasen (*KCNT1*-associated epileptic encephalopathy) and Atipeksen (ATM-related ataxia-telangiectasia) further operationalized and confirmed that individualized ASO therapy can achieve measurable molecular and clinical benefit within months of variant identification following the FDA’s flexible IND model. Each followed a rapid design-validation-toxicology pipeline: patient-derived fibroblast modeling, computational off-target screening, short-term murine safety studies, and intrathecal administration under adaptive dosing. Cumulatively, these programs demonstrated the feasibility of personalized interventional genetics—treating a molecular diagnosis rather than a clinical category [51].

### 4.2. Feasibility of N-of-1 Treatments

Feasibility of N-of-1 ASO development can be evaluated through four intersecting domains—genetic, technical, ethical, and clinical [52]. Genetically, the variant must be well-characterized and mechanistically targetable, often a splice-site or gain-of-function defect in a severe, untreatable condition. Technically, ASO design must demonstrate robust correction or knockdown in vitro using patient-derived cells, with validated potency, minimal off-target activity, and manufacturability under GMP conditions. Ethically, informed consent, transparent risk–benefit analysis, and independent institutional review board oversight are essential. Clinically, feasibility requires a realistic therapeutic window, accessible target tissue, and measurable biomarkers to monitor efficacy and safety [50,52].

Typical preclinical pipelines include in silico prediction, in vitro proof-of-mechanism, and condensed 3-month toxicology studies in rodents. Manufacturing and analytical testing are completed in parallel to minimize delay. Dosing is individualized—often starting with a low-dose bolus followed by escalation guided by tolerability and biomarker responses. Long-term follow-up extends beyond 12 months, emphasizing safety and efficacy monitoring tailored to the target tissue. For CNS indications, this includes CSF analysis and neurofilament light chain quantification in blood or CSF to detect neuroaxonal damage. Together with longitudinal imaging, these coordinated evaluations are essential to determine the safety, biodistribution, and durability of the intervention. These coordinated steps define a reproducible, ethically grounded template for personalized ASO deployment [53,54].

### 4.3. Off Target Effects in N-of-1 Treatments

ASO therapies can produce off-target effects through both hybridization-dependent and hybridization-independent mechanisms [55,56]. Hybridization-dependent off-targeting arises when an ASO binds partially complementary RNA transcripts other than its intended target [57]. Even limited sequence complementarity—particularly within short contiguous regions—can be sufficient to induce unintended RNase H–mediated degradation or aberrant splice modulation. Such interactions may lead to downregulation of non-target genes or unintended exon skipping, potentially disrupting cellular pathways unrelated to the primary disease mechanism. These effects are especially relevant in tissues with high transcriptomic complexity, where low-abundance off-target transcripts may still have critical regulatory functions [57,58].

Hybridization-independent effects are largely driven by the chemical properties of the ASO backbone and sugar modifications rather than sequence complementarity. Phosphorothioate backbones, commonly used to enhance nuclease resistance and cellular uptake, can bind non-specifically to plasma proteins, intracellular proteins, and components of the coagulation and complement systems [55,58]. These interactions have been associated with thrombocytopenia, complement activation, and altered coagulation parameters in some patients. Such effects reflect class-related pharmacologic properties rather than target-specific biology and therefore require careful dose optimization and safety monitoring [59].

Immune stimulation represents another important category of off-target activity. Certain ASO sequences or motifs can activate innate immune receptors, including Toll-like receptors, leading to cytokine release and inflammatory responses. Although chemical modifications such as 2′-O-methyl or 2′-O-methoxyethyl substitutions reduce this risk, immunostimulatory potential remains sequence- and chemistry-dependent. In CNS delivery, intrathecal administration may further influence inflammatory signalling, underscoring the need for careful preclinical immunotoxicity assessment [55,57,58,59].

### 4.4. Ethical and Societal Considerations

While the N-of-1 approach exemplifies the potential of precision medicine, it challenges traditional paradigms of equity and sustainability. The estimated per-patient cost of USD 1–3 million—including oligo synthesis, preclinical testing, and clinical monitoring—poses significant barriers to broad accessibility [54]. Non-profit initiatives such as the n-Lorem Foundation aim to democratize individualized ASOs by providing free drug development for patients with ultra-rare diseases under open-data principles [60,61]. Meanwhile, regulators and bioethicists emphasize transparent data sharing, standardized outcome reporting, and public oversight to ensure accountability and reproducibility.

Ethical implementation also requires long-term post-treatment monitoring to detect delayed toxicities or waning efficacy. Given the absence of large-scale trial data, every N-of-1 case contributes uniquely to collective learning. Efforts are underway to integrate clinical data into shared registries such as TREAT-NMD, enabling evidence synthesis across individualized programs [62]. Ultimately, the success of this paradigm depends not only on molecular correction but also on the creation of equitable frameworks for prioritization, consent, and follow-up.

## 5. Challenges and Outlooks

Despite remarkable progress, several scientific and systemic challenges continue to limit the widespread application of ASO and N-of-1 therapies. Addressing these barriers will determine whether personalized RNA medicine can evolve from rare experimental interventions into a scalable clinical paradigm.

### 5.1. Delivery and Durability

Efficient and sustained delivery of ASOs remains the foremost technical obstacle. Because these large macromolecules—ranging from anionic phosphorothioates to neutral morpholinos—are inefficiently internalized by cells and often sequestered in endosomes, achieving therapeutically relevant concentrations in target tissues remains difficult. This challenge is particularly acute in extrahepatic tissues such as skeletal and cardiac muscle, where spontaneous uptake is markedly limited. While systemic PMOs have demonstrated safety, their bioavailability is limited, necessitating frequent high-dose infusions [63].

Emerging PPMOs and antibody-linked or lipid nanoparticle formulations offer improved uptake, endosomal escape, and tissue selectivity. For example, DG9-conjugated PMOs achieve markedly enhanced exon skipping and dystrophin restoration in both skeletal and cardiac muscle, addressing a major limitation of earlier chemistries [34]. However, further validation is required before these next-generation carriers can be broadly deployed. Long-acting formulations and controlled-release nanoparticles are also being investigated to reduce the burden of chronic administration [64].

### 5.2. Timing and Therapeutic Window

ASO therapies stabilize but cannot reverse established cellular degeneration. Consequently, therapeutic success depends heavily on the timing of intervention [63]. Early administration—ideally before significant neuronal or muscular loss—yields better outcomes, as illustrated by the differential responses in Milasen and Atipeksen cases. This dependency highlights the need for early genomic diagnosis through expanded newborn screening and rapid-turnaround sequencing to identify treatable pathogenic variants during pre-symptomatic stages. Integration of NGS and RNA-seq into standard diagnostic workflows will be essential for maximizing the window of therapeutic opportunity [65].

### 5.3. Economic and Regulatory Barriers

The individualized nature of N-of-1 ASOs imposes high financial and logistical demands. Development costs—including oligo synthesis, condensed toxicology, and clinical monitoring—often exceed USD 1–3 million per patient. Although the FDA’s 2024 guidance for individualized ASOs introduced flexible, proportionate oversight, international harmonization remains limited. Divergent regulatory standards between North America, Europe, and Asia hinder global access and data comparability [60].

Non-profit and hybrid models, such as the n-Lorem Foundation, have emerged to address inequity by providing free ASO development for ultra-rare cases under open-science principles [61]. Broader adoption of such frameworks, coupled with shared manufacturing pipelines and standardized safety datasets, could substantially lower entry barriers and ensure ethical accessibility worldwide.

### 5.4. Data Integration and AI-Driven Design

Standardization of clinical data and interoperability across registries remain critical for collective progress. Global platforms such as TREAT-NMD are beginning to aggregate molecular, safety, and outcome data from rare-disease ASO trials [62]. Harmonized data structures will enable meta-analysis and facilitate evidence-based refinement of future designs.

Meanwhile, artificial intelligence (AI) is emerging as a transformative tool for oligonucleotide engineering. Machine-learning models can now predict binding affinity, splicing outcomes, and off-target effects with increasing accuracy, dramatically accelerating design–test cycles. When integrated with high-throughput in vitro validation and cloud-based oligo libraries, AI-guided pipelines could democratize ASO development, allowing rapid iteration of patient-specific candidates within weeks [66].

### 5.5. Outlook

The next decade will likely witness the transition from bespoke N-of-1 interventions to platform-based “N-of-few” therapeutics, serving small genetic subpopulations using modular chemistries and standardized manufacturing. Convergence of NGS diagnostics, AI-assisted design, and advanced delivery systems will enable rapid, reproducible development of safe and effective ASOs [49].

Equally important will be the establishment of global ethical and regulatory frameworks that balance innovation with equity. Sustainable reimbursement models, transparent data sharing, and long-term safety surveillance must evolve in parallel with technological advances [66]. Through these integrated efforts, the once-extraordinary N-of-1 paradigm may ultimately become a routine component of precision genomic medicine—turning diagnosis into therapy for even the rarest of genetic diseases.

## 6. Conclusions

The convergence of NGS and ASO technologies has ushered in a new era of precision genomic medicine. Taken together, the evidence discussed underscores that this integration is not solely technological progress, but a conceptual shift in how rare genetic diseases are approached. High-resolution genomic and transcriptomic diagnostics now enable rapid identification of pathogenic variants and direct translation into patient-specific RNA therapeutics. From exon skipping in DMD to individualized interventions such as Milasen, Valeriasen, and Atipeksen, these breakthroughs demonstrate that tailored ASOs can restore or modulate gene function within clinically meaningful timeframes.

Nevertheless, key challenges remain in efficient delivery, long-term durability, cost, and equitable access. Continued innovation in peptide, antibody, and nanoparticle-based delivery, AI-assisted oligo design, and platform manufacturing will be critical to expanding the therapeutic reach of ASOs. Equally vital are harmonized regulatory frameworks and ethical oversight to ensure that rapid innovation remains patient-centered and globally accessible, particularly as N-of-1 and ultra-rare applications challenge traditional evidentiary standards and reimbursement models.

As NGS-guided ASO pipelines mature, the boundary between diagnosis and therapy will continue to dissolve—transforming rare, previously untreatable disorders into candidates for individualized RNA correction. This synthesis of genomic insight and targeted molecular intervention marks a pivotal shift toward a future where personalized RNA medicine becomes not an exception, but a standard of care for rare genetic diseases.

## Data Availability

No new data were created or analyzed in this study.
